# Rootstock microbiome as a target for manipulation to combat apple replant disease

**DOI:** 10.1038/s41598-025-05837-w

**Published:** 2025-07-02

**Authors:** Svetlana N. Yurgel, Nivethika Ajeethan, Shawkat Ali

**Affiliations:** 1 Grain Legume Genetics and Physiology Research Unit, USDA, ARS, Prosser, WA 99350 USA; 2 Department of Plant, Food, and Environmental Sciences, Faculty of Agriculture, Dalhousie University, Halifax, NS B2N 5E3 Canada; 3 Kentville Research and Development Centre, Agriculture and Agri-Food Canada, Kentville, NS B4N 1J5 Canada

**Keywords:** Apple replant disease, Apple rootstock, Bacterial microbial communities, Fungal microbial communities, Microbial communities, Agroecology

## Abstract

Apple replant disease (ARD) describes a phenomenon of reduction of crop productivity in the early years of orchard establishment on sites previously planted with apple. Currently, manipulation of the soil microbiome through (bio)fumigation is the primary approach to alleviate ARD. An alternative approach to combat ARD, could involve adjusting the rootstock microbiome to better cope with biotic stress present in orchard soil. In this study we evaluated differences in microbiome structure and composition between nursery grown rootstock and mature apple trees, cultivated in Nova Scotian orchards. We found that mature apple tree roots associated microbiome dramatically differed in its diversity, structure and composition compared to that associated with saplings. Our research identified several fungal and bacterial taxa as potential candidates for further study in the context of nursery inoculation and their possible role in mitigating ARD in re-planted apple orchards. The results of this study provide a foundation for development of a synthetic community which could be used in nurseries during rootstock propagation to improve saplings adaptation to ARD soils. This approach may offer an ecologically safe and cost-effective alternative to current soil amendments to alleviate ARD consequences.

**Supplementary Information:**

The online version contains supplementary material available at 10.1038/s41598-025-05837-w.

## Introduction

Replant disease describes a phenomenon of reduction of crop productivity due to pathogens accumulation in fields when the same or closely related plant species are repeatedly planted in the same field. It can affect a broad spectrum of plants, including perennials such as apples, pears, plums, cherries and grapes^1^. Apple replant disease (ARD) symptoms include poor establishment and growth of the new planting resulting in crop losses in the early years^2^. The disease is caused by a complex of pathogens including nematodes, fungi and oomycetes. However, actual relative abundance of the pathogens and their interaction in the specific site might be influence by abiotic factors such as soil moisture and temperature^3^. Soil fumigation is the dominant way to control ARD in the USA. Other methods of management include application of soil amendments^4–8^, alteration of the soil microbiome, and utilization of disease tolerant rootstocks.

The main strategies for manipulation of soil microbial community continue to be pre-plant application of Brassicaceae seed meal^9^, or bio-fumigation, which affects both soil and plant rhizosphere microbiomes. An incorporation of Geneva and Malling series apple rootstocks in the field studies indicated that one of the factors affecting the efficiency of bio-fumigation is the rootstock genotype^6,10^. Specifically, Geneva rootstocks exhibited a better response to bio-fumigation than Malling. It has been proposed that differential recruitment of plant-beneficial microbes to the rootstocks rhizosphere contributes to the greater protective effect of the bio-fumigation on Geneva rootstocks^10^.

Only a few studies have investigated the role of root-associated microorganisms in ARD^11–14^. The root-associated microbiome, which originates from the orchard soil and reflects previous plant history, undergoes a strong selective pressure driven by from environmental stresses and plant genotype^3,15^. Ideally, this microbiome helps protect the plant from biotic and abiotic stresses, such as pathogenic infections^16^. We hypothesize that the young trees originating from nurseries lack root-associated microorganisms that could help them withstand environmental stresses present in the orchard soils. Therefore, introducing a synthetic community isolated from the roots of mature, healthy tree roots might help “immunize” young trees and improve ARD tolerance. However, very limited information is currently available on the microbiomes associated with apple sapling roots during nursery propagation^17^. To our best knowledge, no research has been conducted to assess the differences between apple saplings and mature tree root microbiomes to assess validity of this hypothesis.

The objectives of this research were: (1) to evaluate the differences in microbiomes associated with mature tree roots and the ten most commonly used apple rootstock, obtained directly from a single nursery; (2) to identify root-associated microorganisms that may play a role in plant protection against ARD pathogen complex that develops in old apple orchards after prolonged tree cultivation; and (3) to assess the effect of rootstock genotype on microbiome assembly during nursery propagation.

## Results

### Overall microbiome composition

In total, 1,815 fungal and 5,739 bacterial amplicon sequence variants (ASVs) were identified in the datasets. Most abundant bacterial and fungal genera found in rootstock were *Allorhizobium-Neorhizobium-Pararhizobium-Rhizobium* group (13% 16 S reads), *Sphingobium* (8% 16 S reads), *Streptomyces* (7% 16 S reads), *Pseudomonas* (7% 16 S reads), unannotated Helotiales (12% ITS reads), *Hyaloscypha* (10% ITS reads), and *Tetracladium* (7% ITS reads), while the tree microbiome was populated with bacteria *Streptomyces* (7% 16 S reads), *Niastella* (4% 16 S reads), *Kineosporia* (4% 16 S reads), and fungi *Pteridiospora* (53% ITS reads) and unannotated Helotiales (12% ITS reads).

### Microbiomes associated with the mature tree roots differ from the rootstock microbial communities

#### Rootstock and tree microbiomes differ in their alpha- and beta-diversity

**Fig. 1:**
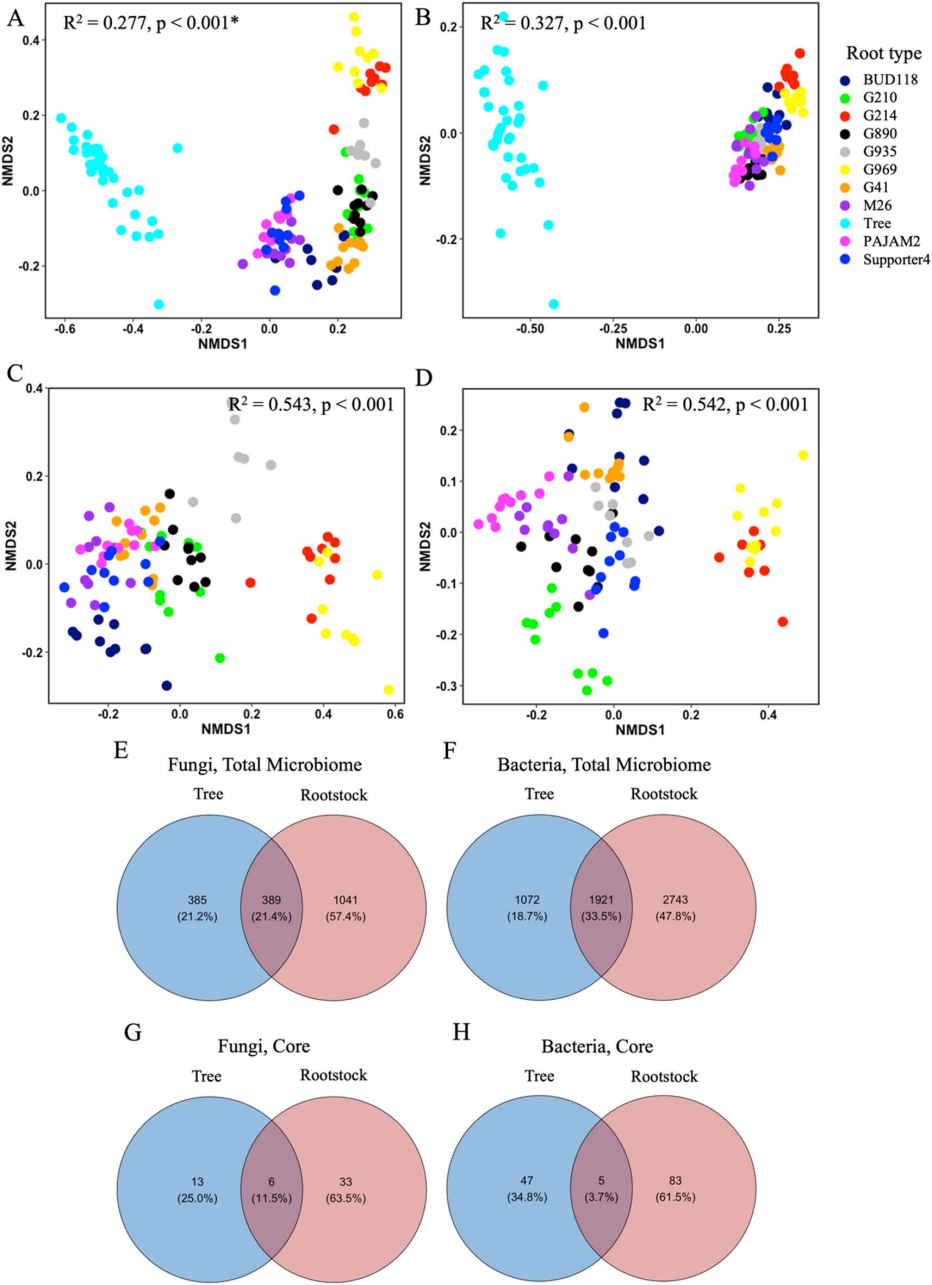
Overall structure of tree and rootstock microbiomes. **A**-**D** – Nonmetric multidimensional scaling (based on Bray-Curtis dissimilarity distances) of bacterial and fungal communities at the ASV level; **A**, **C** – ITS; **B**, **D** – 16 S rRNA. **A**, **B** – Tree and rootstock microbial communities. **C**, **D** – Rootstock microbial communities. *Adonis tests were used to assess whether beta-diversity is related to sample groupings, 999 permutations. **E**-**H** – Venn diagrams showing overlap in ASVs between niches; Overlap in **E** – Total fungal and F – total bacterial ASVs; **G** – Core fungal and **H** – core bacterial ASVs.

**Fig. 2:**
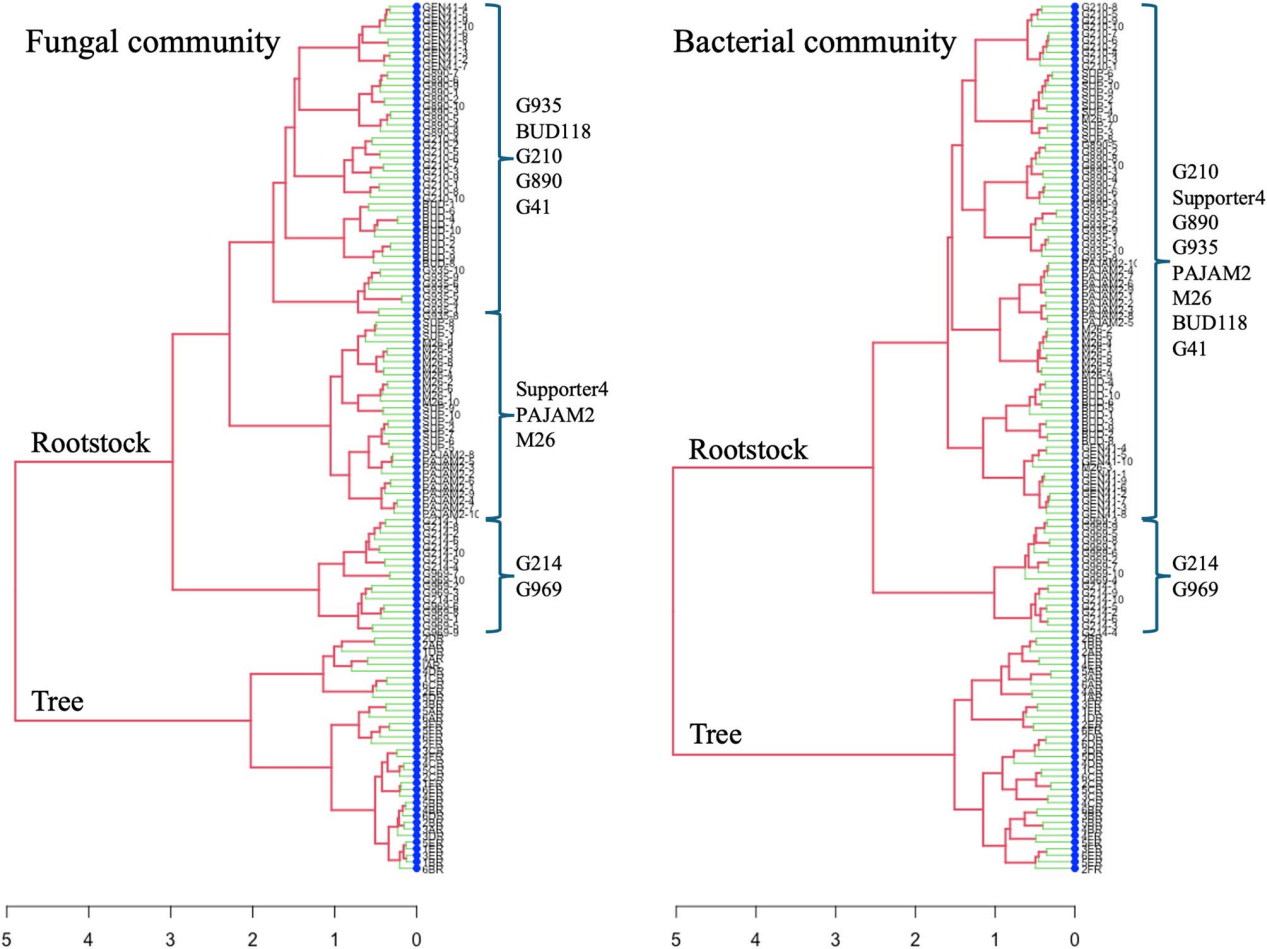
Hierarchical clustering of the fungal community and bacterial community.

The origin of the microbiome (tree vs. rootstock) of both fungal and bacterial communities was a significant factor that shaped microbiome structure. Around 28% of fungal and 33% of bacterial community variation was explained by the origin of the microbiome (Fig. 1; Table 1). These differences were well visualized on NMDS plots (Fig. 1A, B) showing distinct clusters formed by tree and rootstock microbial communities. Similarly, a hierarchal clustering analysis identified strong separation based on the origin of microbiomes, which was true for both fungi and bacteria (Fig. 2). The tree samples formed a single cluster distantly separated from the rootstocks, indicating major differences derived from microbiome origin. A larger number of bacterial and fungal ASVs were identified in the rootstock, compared to the tree microbiome. More specifically, close to twice as many fungal and bacterial ASVs were detected in rootstocks compared to tree roots, 1,430 vs. 774 and 6,4642 vs. 993, respectively (Fig. 2, E, F), which might be attributed to a large set of rootstock samples compared to tree samples. Fungal alpha-diversity was more affected by the microbiome origin compared to bacterial alpha diversity. Fungal Shannon entropy was significantly (t-test *p* < 0.001) lower in the tree than in the combined rootstock microbiome, 2,8 vs., 5.5, respectively, while bacterial Shannon diversity did not differ significantly (t-test *p* > 0.05) based on the microbiome origin, 7.6 vs. 7.8 in tree and rootstock microbiomes, respectively.

**Table 1:** Variation in sample groupings as explained by weighted Bray-Curtis dissimilarity distances.

Grouping	ITS, R2	16 S rRNA, R2
Origin (rootstock vs. mature tree root)	0.277***	0.327***
Saplings		
Rootstock	0.543***	0.542***

Adonis tests were used to assess whether beta-diversity is related to sample groupings, 999 permutations, R2, *P < 0.05, **P < 0.01, and ***P < 0.001.

#### Rootstock and tree microbiomes differ in their composition

**Fig. 3:**
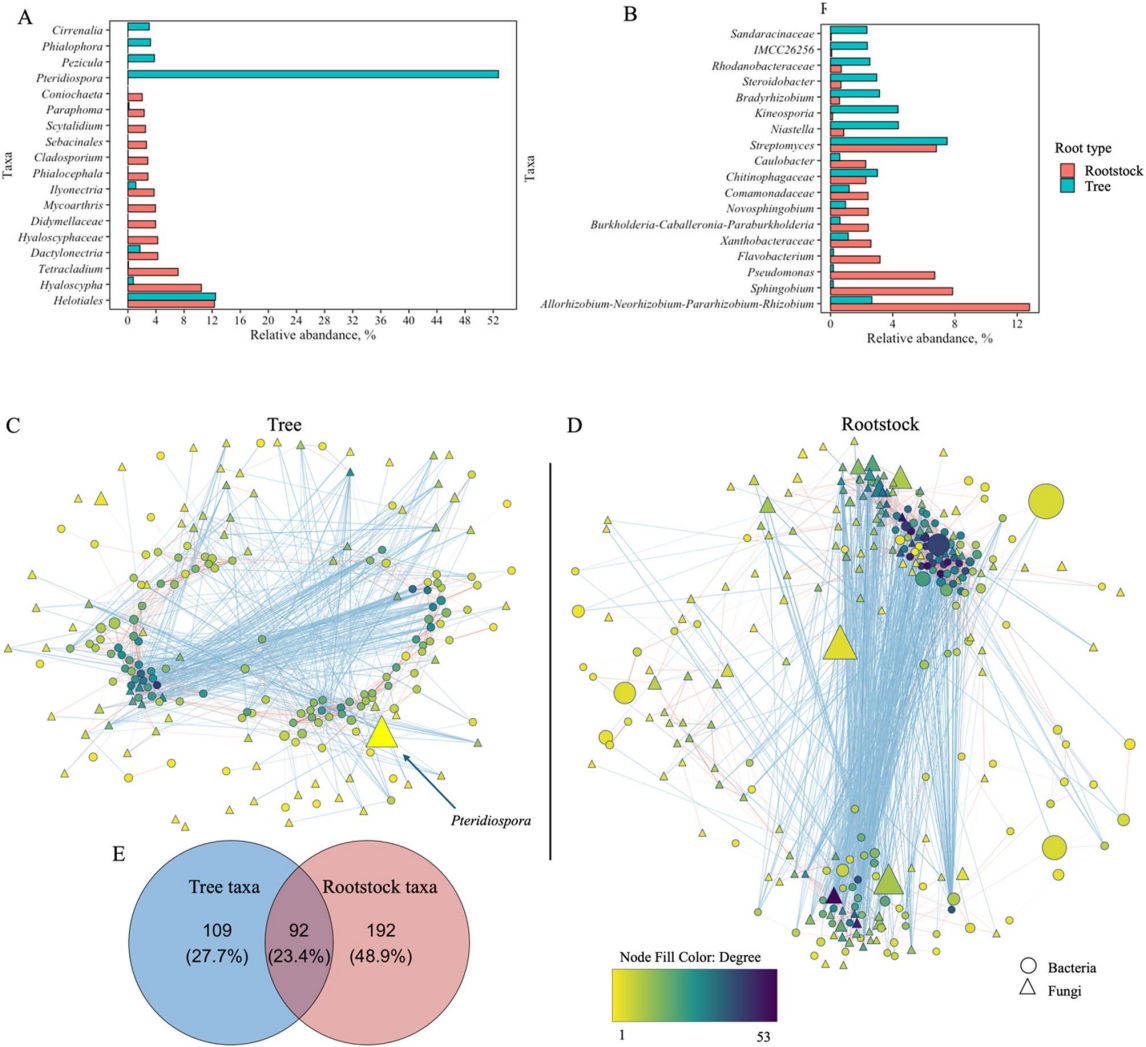
Structural and interactional differences between rootstock and tree microbiomes. **A**, **B** – Taxa differentially represented tree and rootstock microbiomes; A – Fungal and B – Bacterial genera. Only taxa represented by at least 1% of total 16 S ribosomal RNA or internal transcribed spacer reads in at least one group are shown, at genera level annotation; Expected Benjamini-Hochberg corrected *P* value of Welch’s t-test, wi.eBH < 0.05. C-E – Co-occurrence networks generated by measuring abundance co-correlation between microbial genera; **C** - Tree and **D** – Rootstock microbiome. Correlation base network analysis showing potential interactions between bacterial and fungal genera. The size of the node is proportional to a taxon’s average relative abundance across all the samples. The lines connecting nodes (edges) represent positive (red) or negative (blue) co-occurrence relationship. The intensity of the color and the length of the edges represent the strength of correlation. E - Venn diagrams showing overlap in genera identified in tree and rootstock co-occurrence network.

Close to 50% of fungal and bacterial taxa with annotation at a genus level were differentially represented between the tree and rootstock microbiomes. More specifically, 406 fungal and 592 bacteria genera were identified in our study, out of which 193 fungal and 274 bacterial genera differ in their relative abundances between the niches (Table S1; Table S2). These genera were represented by the majority of the reads in both microbiomes. The differentially distributed fungal genera were represented by 90% and 94% of total ITS reads, and the bacterial genera were represented by 85% and 83% of total 16 S rRNA reads of the rootstock and tree microbiomes, respectively. The most abundant genera, represented by at least 2% of total ITS and 16 S rRNA reads and differentially represented between the rootstock and tree microbiomes are shown on Fig. 3, A and B.

The fungi *Pteridiospora* was highly abundant in tree roots with a total relative abundance of 53%, while it comprised 0.02% of ITS reads in rootstocks (Fig. 3, A). A single ASV 3913eeb46ce3691b0f8e4c83972169e9 was represented by 51% of tree root ITS reads. The ASV was manually annotated as *Pteridiospora spinosispora*. Additionally, *Pezicula*, *Phialophora*, and *Cirrenalia* were more relative abundant in the tree compared to root stock microbiome. On the other hand, taxa containing potential symbiotic and pathogenic fungi such as *Hyaloscypha*, *Dactylonectria*, *Ilyonectria*, *Phialocephala*, *Cladosporium*, and *Sebacinales* were higher in relative abundances in the rootstock compared to tree microbiome.

The most relatively abundant bacterial genera in total tree and rootstock microbiomes, combined, *Streptomyces* was significantly more relatively abundant in tree roots, although the actual difference in its relative abundance between the tissue was minor, 6.8% and 7.5% in tree and rootstock tissue, respectively (Fig. 3, B). Several taxa containing plant growth promoting bacteria, including *Niastella*, *Kineosporia*, *Bradyrhizobium*, and *Steroidobacter*, had significantly higher relative abundances in the tree combated to rootstock microbiome, while *Caulobacter*, *Sphingobium*, *Allorhizobium-Neorhizobium-Pararhizobium-Rhizobium* group, *Pseudomonas*, *Flavobacterium*, *Burkholderia-Caballeronia-Paraburkholderia* group were overrepresented in rootstocks.

### Tree and rootstock core microbiome

The core ASVs were defined as present in at least 95% of samples in each group. We identified 19 and 39 fungal ASVs in tree and rootstock core microbiome, respectively (Fig. 1G; Table S3). These ASVs were represented by more than 74% and 44% of tree and rootstock ITS reads, respectively. Six ASVs were part of both the tree and rootstock fungal core microbiome and were represented by nearly 62% and 17% of tree and rootstock total ITS reads, respectively. *Pteridiospora* ASV 3913eeb46ce3691b0f8e4c83972169e9 was the most abundant in the tree core microbiome but only represented by 0.02% of rootstock total ITS reads.

The bacterial core microbiome contained 52 and 88 tree and rootstock ASVs, respectively (Fig. 1H; Table S3). Five bacterial ASVs, annotated as *Bradyrhizobium*, *Dongia*, Xanthobacteraceae, *Labrys soli*, and Microscillaceae were found in both core microbiomes. However, they were of relatively low abundance and represented by < 2% of tree and rootstock 16 S rRNA reads each. In contrast to the fungal tree core microbiome, which was comprised by a substantial number of total tree ITS reads, the bacterial core tree microbiome was represented by 27% of 16 S rRNA reads.

#### Microbiome in the rootstock tissue exhibited stronger co-occurance than in the tree root

Analysis of interkingdom cooperation showed that the rootstock microbiome exhibited stronger co-occurance, compared to the tree microbial communities. That was reflected in a higher number of nodes and average number of neighbors, as well as in increased nodes Betweenness Centrality and Radiality in the rootstock, compared to the tree microbial community (Fig. 3, C, D; Table 2; Table S4; Table S5). We did not detect a significant overlap between the taxa comprising the tree and rootstock cooperation network. Less than half of taxa comprising the tree cooperation network were also found in the rootstock cooperation network and 68% of taxa found in rootstock cooperation network were unique to this tissue (Fig. 3, E). Based on taxa Degree (a number of direct connections), ClosenessCentrality, Radiality and Stress characteristics (indicators of regulatory relevance of the node in the network^18^), bacteria *Pseudoxanthomonas* and *Taibaiella* were the most influential taxa in the tree co-occurrence network. Fungus *Pteridiospora* was a part of the tree network, although, based on its network parameters, it did not have strong involvement in overall microbial co-occurance. Based on Degree, ClosenessCentrality, Radiality and Stress characteristics, fungi Hyaloscyphacea and *Penicillium* were the most influential taxa in the rootstock co-occurrence network.

**Table 2:** Network and node statistics.

	Rootstock	Tree
Number of nodes	284	201
Number of edges	2017	1075
Network diameter	7	8
Network radius	4	4
Characteristic path length	3.063	3.060
Clustering coefficient	0.366	0.363
Network density	0.050	0.055
Network heterogeneity	0.912	0.791
Network centralisation	0.138	0.164
Avg. number of neighbors***	14.204	10.696
BetweennessCentrality***	0.007	0.010
Radiality***	0.961	0.952

The parameters indicated by brackets are significantly different according to T–test. *** – *p* < 0.001.

## Rootstock and tree microbiome differ in their functional capabilities

**Fig. 4:**
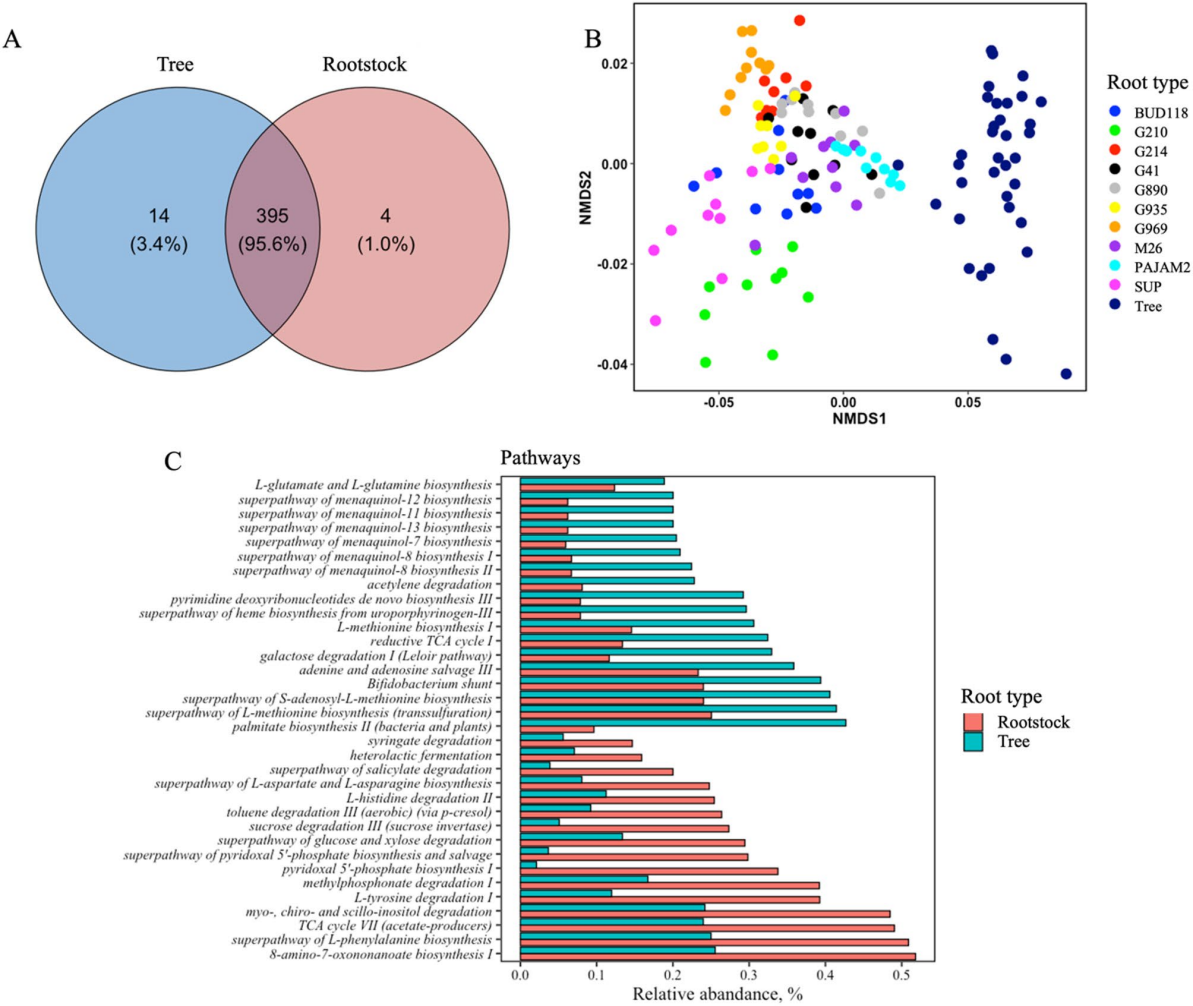
Functional capabilities of tree and rootstock bacterial community. **A** – Venn diagrams showing overlap in pathways identified in tree and rootstock microbiome. **B** – Nonmetric multidimensional scaling (based on Bray-Curtis dissimilarity distances) of bacterial communities based on identified pathways. *Adonis tests were used to assess whether beta-diversity is related to sample groupings, 999 permutations. **C** – Relative abundances of pathways differentially represented between tree and rootstock microbiomes. Only most abundant pathways having at least 2-fold differences between the tissues are shown. Expected Benjamini-Hochberg corrected *P* value of Welch’s t-test, wi.eBH < 0.05.

Prediction of metagenome functional content from 16 S rRNA gene identified 413 metabolic pathways in combined tree and rootstock microbiomes (Fig. 4, A). 395 pathways were common to both root types, 14 were unique to the tree and 4 to the rootstock microbiomes. Even though most of pathways were identified in both microbiomes, Adonis test indicated that 54% (*p* < 0.001) of variations between the tree and rootstock microbiome functions was explanined by origin of community. The difference between structure of these functional capabilities was well visualized on NMDS plot (Fig. 4, B). The majority, 367, of pathways identified in the study were differentially represented between the tree and rootstock microbiomes. These pathways are listed in Table S6. The most abundant pathways, which were higher in relative abundances in one of the tissues at least two times are shown on Fig. 4, C. Several menaquinol and methionine biosynthetic pathways were more relatively abandant in the tree compared to rootstock microbiome. On the other hand, degradation pathways, including degradation of compounds with plant and industrial origin, such as inositol, syringate, salicylate, methylphosphonate, and toluine were relatively abundant in the rootstock compared to tree microbiom.

### There were significant variations between the sapling’s rootstock microbiomes diversity and composition

**Fig. 5:**
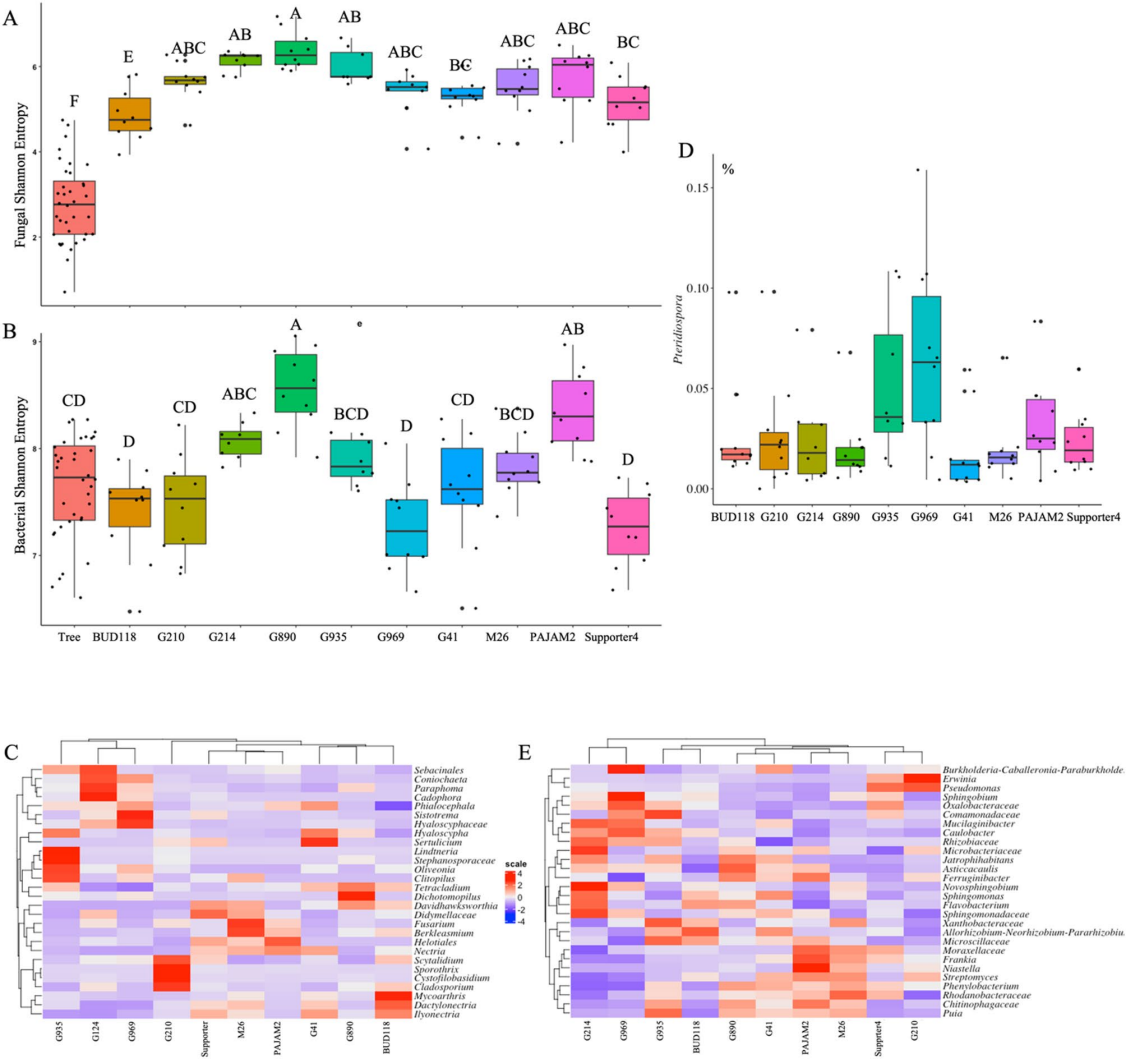
Variations in microbial community across rootstocks. **A**, **B** – Microbial alpha-diversity For each variable, data followed by different letters are significantly different according to Kruskal-Wallis pairwise test (*p* < 0.05). Corrected p-values were calculated based on Benjamini-Hochberg FDR multiple test correction. Capital letters indicate differences between root types. **C**-**E** – Relative abundances of microbial taxa differentially represented between rootstocks. **C**– fungal taxa represented by at least 5% of rootstock ITS reads; **D** – Pteridiospora, **E** – bacterial taxa represented by at least 1% of rootstock 16 S rRNA reads. Expected Benjamini-Hochberg corrected P value of Kruskal-Wallace test, kw.eBH < 0.05.

Bacterial and fungal microbiomes vary significantly across rootstocks. The rootstock contributed to 54% of both fungal and bacterial community variation (Fig. 1, C, D; Table 1). These rootstock microbiomes formed separate clusters based on fungal and bacterial hierarchal clustering analysis (Fig. 2). Additionally, communities from rootstocks G969 and G214 were closer to each other and more distant from the rest of the rootstock communities in their structure (Figs. 1 and 2). We also detected some differences in microbial alpha-diversity across rootstocks compared to the tree microbiome. The fungal Shannon entropy was significantly higher in all individual rootstocks, compared to tree microbiome (Fig. 5A), and microbiomes from BUD118 exhibited the lowest Shannon entropy. On the other hand, in most rootstocks, bacterial alpha-diversity communities did not differ significantly from tree microbiome (Fig. 5B). The only significant increase in bacterial Shannon entropy was detected in G890 and PAJAM2 rootstock.

#### Composition of fungal and bacterial microbiomes differ significantly across rootstocks

375 fungal and 245 bacterial genera were differentially represented across rootstock microbiomes (Table S7; Table S8). The relative abundances of 28 fungal and bacterial genera represented by at least 5% of ITS and 1.5% of 16 S rRNA reads, respectively, are shown on Fig. 5, C, D. Hyaloscypha was one of the most relatively abundant genera in G14 (24% of ITS reads), G935 (24% of ITS reads), and G210 (11% of ITS reads). It was also the second most abundant taxon in G890 (14% of ITS reads). Unannotated Helotiales was the most abundant taxon in Pajam2, Supporter and M26, comprising 31%, 22% and 16% of ITS reads in corresponding rootstock. Bud118 microbiome had higher relative abundances of *Mycoarthirs* (17% of ITS reads) and *Dactylonectria* (11% of ITS reads), compared to other rootstocks. Unannotated Hyaloscyphaceae was the most abundant taxa in G214 (10% of ITS reads) and G969 (19% of ITS reads) (Fig. 5, C; Table S7). While of relatively low abundance, genera *Pteridiospora* was differentially represented between rootstocks, it was relatively most abundant in rootstocks G969 and G935 (Fig. 5, D; Table S7). The most prominent genera in rootstock bacterial communities were *Allorhizobium-Neorhizobium-Pararhizobium-Rhizobium* group, *Sphingobium* and *Pseudomonas*. *Allorhizobium-Neorhizobium-Pararhizobium-Rhizobium* group was the most relatively abundant in Bud118, G214, G890, G935, G41, Pajam2, and Supporter. *Sphingobium* and *Pseudomonas* were overrepresented in G969 and G210, respectively (Fig. 5, E; Table S8).

## Discussion

All multicellular eukaryotic organisms associate with microbial and viral communities to form ecological units known as holobionts^19^. While specific interactions may differ among organisms, there are fundamental principles that govern how holobionts begin, develop, adapt and maintained. Perennial plants, such as fruit trees, serve as models of holobionts, for studying plant-microbiome co-evolution, particularly in relation to their ability to resist environmental stresses. In this study, we evaluated the root microbiomes of 10 apple rootstocks commonly used in Nova Scotia (NS), Canada, including six Geneva, one Malling and three of European origin, as well as the roots of 36 mature apple trees from six orchards across the province^20^. The rootstock material was obtained directly from NS nursery supplier, and according to the nursery, there were no differences in propagation practices (e.g., tissue culture or layer beds establishment) across genotypes. This implies that all rootstocks were subjected to similar biotic and abiotic conditions, minimizing environmental variations in root-associated microbiome. The selection of ten diverse rootstock genotypes was intended to reduce genotype bias in microbiome assembly, enabling meaningful differences between mature tree roots and rootstocks microbiome potentially contributing to ARD tolerance. The mature tree root (tree) microbiome was obtained from 100- and 30-year-old apple orchards, which provided a solid baseline for apple root microbiomes adapted to biotic and abiotic conditions typical of long established apple orchards.

Mature apple tree roots possessed a microbiome that dramatically differed in diversity, structure and composition from those associated with saplings. This was reflected in (i) a significant effect of origin factor (tree vs. rootstock) on overall community structure; (ii) strong hierarchical clustering that separated tree and rootstock microbiomes into two clusters; (iii) differences in the intensity of microbial interactions between the two groups; and (iv) a large number of differentially represented taxa across the two niches. Our data indicated less complex microbial interaction in tree, compared to rootstock microbiomes, as reflected in decreased number of taxa involved in the interaction and fewer connections among them^21–23^, potentially pointing to decreased multifunctionality within the mature tree microbiome^24^. Moreover, both tree and rootstock microbial networks incorporated a significant proportion of taxa unique to each niche, probably caused by specification to the unique orchard soil environment. This interpretation is supported by lower Shannon diversity observed in tree fungal communities, compared to rootstock^25,26^, and by strong differences between the two microbiomes function.

A large number of bacterial and fungal genera were differentially represented between rootstock and tree microbiomes. Among them, several taxa associated with potential root rot pathogens, such as *Dactylonectria*, *Ilyonectria*, *Phialocephala*, *Cladosporium*, and *Sebacinales*, had higher relative abundances in the rootstock, compared to tree microbiome^27–30^. If pathogenic, these taxa may contribute to disease development in the saplings making them more susceptible to ARD. *Pteridiospora spinosispora* was the most relatively abundant species in tree fungal community, it comprised of more than 50% of total tree ITS reads. On the other hand, it was of relatively low abundance in rootstocks. Despite this difference, it was a part of the core fungal microbiome, found in 95% of both tree and rootstock samples.

Several fungal and bacterial taxa with potential plant growth promoting traits^31–34^ – such as fungi *Pteridiospora*, *Pezicula*, and *Phialophora* and bacteria *Niastella* and *Bradyrhizobium* – were significantly enriches in the mature roots compared to rootstocks. In a previous study *P. spinosispora* was identified as one of two taxa inversely correlated with ARD tolerance^17^. In the current study, this species was found in high relative abundance in the tree roots across all six locations, highlighting its potential universal role in supporting tree health under orchard soil conditions in NS. Our data underscore the need for further investigation into the potential beneficial role of *P. spinosispora* other bacterial and fungal taxa that are overrepresented in the mature tree roots, compared to rootstocks, in the mitigation of ARD.

In a previous study, *Streptomyces* was detected at very low relative abundance in pre-planted M26 rootstock^11^. Additionally, a negative correlation between the relative abundance of *Streptomyces* and plant growth parameters was detected, implying a potential role of this genus as an opportunist or part of the ARD complex. These findings were in contrast with our data showing a significant presence of this genus in all rootstocks including M26. We also detected a minor but significant overrepresentation of this genus in tree microbiome, implying a more complex role of *Streptomyces* in ARD tolerance. We found a strong community variation across rootstock genotypes. Many Streptomyces spp. produce antimicrobial compounds, so it is possible that the species observed in this study were ones that helped to prevent ARD. A previous study assessing microbiomes of rootstock after one year of growth in orchard soils reported a strong effect of genotype on the structure of the fungal, compared to the bacterial community^35^. However, the effect of this factor was lower, compared to our study (12% and 5% of fungal and bacterial community variation, respectively, was attributed to rootstock genotype). In comparison our study attributed 54% of both bacterial and fungal community variation to rootstock genotype. These differences could be attributed to the larger sampling set in our study, 10 rootstocks vs. 4 in the previous study^35^. Several other studies have also reported a strong effect of genotype on structure of endophytes, although these were conducted on saplings following orchard field propagation^10,36–38^. Interestingly, G214 and G969 bacterial and fungal community profiles clustered more closely with each other than with other genotypes, including rootstock sharing the same parentage (e.g. G890 and G935 both derived from Robusta 5 x Ottawa 3). This suggests that factors additional to pedigree affecting microbiome assemblage in sapling roots. We did not identify any specific plant characteristics which could be linked to the differences in microbiome sorting between rootstocks and according to the supplier all rootstocks were propagated under the same conditions.

The concept of using synthetic community comprised of root-endophytes to improve host-plant growth and development has been previously tested and proven to be highly efficient^39^. Although limited to microbiome analysis and not aimed at verifying the roles of individual taxa, the results of this study provide a foundation for the developing a synthetic community that could be used in nurseries during rootstock propagation to improve sapling adaptation to ARD soils. This approach could provide a more ecologically safe and cost-efficient alternative to soil amendments to alleviate ARD consequences. In addition to increasing resistance of apple trees to replant disease, such inoculation could potentially improve saplings growth and development and accelerate their maturation and fruit production. Moreover, this approach could be extended to other perennial crops undergoing re-planting problem.

## Methods

### Plant material and samples processing

The material of 10 rootstocks (Table S9) was obtaned through Scotian Gold Cooperative and was originated from Oregon Rootstock and Tree Co. Inc. The rootstocks were produced by layering method. The orginal material was sent to Clean Plant Center Northwest (CPCNW) in Washington State for maintaining and testing all the cultivars for viruses. The tested buds were released back for multiplication through tissue culture and establishing new layer beds. According to the suppliers, there was no difference in the way they used the tissue cultured rootstock to establish layer beds with the different genotypes. However, the tissue culture labs might use different agar recipes per different genotypes (personal communication).

The roots were cut from the rootstocks, placed in sterile bags and transported to the laboratory on ice. The processing of the roots and DNA isolation were done as described previously^20^.

## PCR amplification and illumina sequencing

At least 50 ng (10 µL) of DNA samples were sent to the Dalhousie University Centre for Comparative Genomics and Evolutionary Bioinformatics–Integrated Microbiome Resource (CGEB-IMR) for V6–V8 16 S rRNA gene (16 S; forward: 5′-ACGCGHNRAACCTTACC-3′; reverse: 5′-ACGGGCRGTGWGTRCAA-3′) and ITS2 region (ITS2; forward: 5′-GTGAATCATCGAATCTTTGAA-3′; reverse: 5′-TCCTCCGCTTATTGATATGC-3′) library preparation and sequencing. Samples were multiplexed using a dual-indexing approach and sequenced using an Illumina MiSeq with paired-end 300 + 300 bp reads. The Illumina sequencing and multiplexing was conducted as described in^40^.

## Bioinformatic and statistical analysis

The raw rootstock reads were combined with the raw sequencing data obtained from corresponding mature tree roots (tree) samples^20^. The tree samples comprised microbiomes of 36 mature trees collected from six mature apple orchards located in the Annapolis Valley, Nova Scotia, Canada. The age of orchard ranged from 30 to approximately100 years.

Paired ends raw amplicon sequences were first stitched together using Paired-End reAd mergeR (PEAR)^41^. Cutadapt v1.11 was used to trim primer sequences from the available reads^42^. Trimmed reads were further processed with QIIME 2 version 2022.11^43^. QIIME2’s *q-*score-joined function was used to filter low-quality sequences. The reads were trimed to a length of 253 bp (ITS) and 283 (16 S rRNA). ASVs were classified taxonomically using a Naïve-Bayes RDP classifier and accessing the UNITE ITS^44^ and Silva 16 S rRNA databases^45^. To account for the internal sequencing error of the Illumina MiSeq platform, we removed ASVs with a mean relative abundance of less than 0.01% of the dataset, resulting in the exclusion of ASVs below a threshold of 60 16 S rRNA and 96 ITS reads. Plant-derived ASVs, such as chloroplast or mitochondrial, as well as unannotated ASVs were also removed.

### Data analysis and statistics

After deblurring and filtering 13,083,842 (median frequency 96,204) ITS and 6,173,790 (median frequency 45,395) 16 S rRNA reads were retained in the analysis (Table S9). Three ITS samples with the depth < 6,634 and four 16 S rRNA samples with the depth < 8,384 were removed from the analysis. For alpha-diversity estimation the ITS and 16 S rRNA sets were rarefied to the depth of 6,634 and 8,384 reads, respectively (Table S9).

QIIME2 diversity core-metrics-phylogenetic plugin was used to generate Shannon diversity and β-diversity metrics. Variations in sample groupings explained by Bray-Curtis β-diversity distances (permutational multivariant analysis of variance [PERMANOVA] or Adonis tests, 999 permutations) were run in QIIME2. Nonmetric multidimensional scaling (NMDS) plots and hierarchical cluster analysis were built based on Bray-Curtis distances using the Vegan package in R^46^. NMDS plots were visualized using the gglpot2 package in R^47^. Differential relative abundances for both 16 S rRNA and ITS2 were determined using ALDEx2^48^. The cooccurrence analysis was performed using the CCREPE (compositionality corrected by renormalizaion and permutation) R package^49^. To obtain comparable datasets, 36 replicate samples were selected from all successfully sequenced rootstock samples, Bud118–4, G210–4, G214–4, G890–4, G935–4, G969–4, G41–3, M26–3, PAJAM2–3, and Supporter4–3 samples. The resulted with p-values > 0.01 were filtered to remove non-statistically significant relationships. Network visualization and node and network statistics calculations was done using Cytoscape 3.9.1^50^.

## Electronic supplementary material

Below is the link to the electronic supplementary material.

Supplementary Material 1

## Data Availability

The datasets generated for this study can be found in the NCBI sequence read archive under BioProject IDs: 16 S rRNA – PRJNA1187235 and ITS2 – PRJA1187243.
